# Integration of Tumor Microenvironment in Patient-Derived Organoid Models Help Define Precision Medicine of Renal Cell Carcinoma

**DOI:** 10.3389/fimmu.2022.902060

**Published:** 2022-05-03

**Authors:** Bingran Wang, Yizheng Xue, Wei Zhai

**Affiliations:** Department of Urology, Renji Hospital, School of Medicine, Shanghai Jiao Tong University, Shanghai, China

**Keywords:** patient-derived organoids, renal cell carcinoma, tumor microenvironment, precision medicine, immunotherapy

## Abstract

Renal cell carcinoma (RCC) is a common urological tumor, with a poor prognosis, as the result of insensitivity to chemotherapy and radiotherapy. About 20%–30% of patients with RCC have metastasis at the first diagnosis, so only systemic treatment is possible. Due to the heterogeneity of renal tumors, responses to drugs differ from person to person. Consequently, patient-derived organoid, highly recapitulating tumor heterogeneity, becomes a promising model for high-throughput *ex vivo* drug screening and thus guides the drug choice of patients with RCC. Systemic treatment of RCC mainly targets the tumor microenvironment, including neovasculature and immune cells. We reviewed several methods with which patient-derived organoid models mimic the heterogeneity of not only tumor epithelium but also the tumor microenvironment. We further discuss some new aspects of the development of patient-derived organoids, preserving *in vivo* conditions in patients with RCC.

## Introduction

In recent years, with the advanced knowledge of tumor biological behaviors, especially in the aspect of tumor heterogeneity, tumor treatment became personalized under the guidance of molecular classification (1–3). To further investigate the mechanisms of oncogenesis, tumor invasion, and metastasis and its translation to novel therapies, *in vitro* models such as cell lines are fundamental. However, a significant gap exists between the modeling system and *in vivo* conditions of patients, resulting in the inaccuracy of the predictive ability of several preclinical models. To shorten the gap, patient-derived models, including patient-derived primary tumor cells (PDTCs), patient-derived organoids (PDOs), and patient-derived xenograft (PDX) have been developed as a preclinical model for nearly all kinds of solid tumors (4).

Renal cell carcinoma (RCC) ranks the 6th most commonly diagnosed cancer in men and 10th in women (5). RCC can be majorly divided into three subtypes, including chromophobe (chRCC), papillary (pRCC), and a most common type clear cell RCC (ccRCC), accounting for 75% of cases of RCC (6). In addition, a large number of patients present with metastatic RCC at the time of the first diagnosis, which makes radical surgery infeasible (7). Consequently, here we represent the current knowledge of PDO establishment in patients with RCC and its significance in personalized medicine.

## Evolution of Patient-Derived Organoid Model in Renal Cell Carcinoma

Organoids are 3D cultured cell models that partially preserve the characteristic architecture of organs, for example, crypt structure in intestinal organoids (8) and tubule structure in kidney organoids (9). PDO models are organoids established from tumor tissues resected from patients, which recapitulate hallmarks of parental tumors both histologically and genetically. Immunohistochemistry, whole-exosome sequencing, and RNA sequencing are routinely used to validate that PDO models are highly consistent with parental tumors and retain inter-tumor heterogeneity (10–12). Moreover, single-cell RNA sequencing (scRNA-seq) technology is expected to provide us with a more comprehensive genetic landscape of PDOs to reveal intra-tumor heterogeneity. Kumar et al. applied scRNA-seq to show the transcriptome profiles of PDO models and validate the preservation of intra-tumor sublineage heterogeneity and transcriptional plasticity of parental gastric cancer tissue (13). In addition, the spatial transcriptome platform has the ability to preserve the spatial architecture of PDOs in the process of scRNA-seq, allowing the investigation of interactions between tumor and microenvironment (14). In the future, more detailed validation of PDO models *via* the latest technologies should be emphasized. As a result, biobanks of PDOs with comprehensive multi-omics information will be established, and researchers will benefit from such a database unprecedentedly.

In comparison to PDX, PDO models take less time to cultivate and have a higher success rate, which is feasible for high-throughput drug screening (15). When compared with PDTC, PDO models show the ability to preserve tumor heterogeneity. Consequently, PDO is a precisely predictive model for drug screening, which mirrors the heterogeneity of drug efficacy. In several cancer types, including lung cancer (16), colorectal cancer (CRC) (17), prostate cancer (12), and glioblastoma (18), PDO models have become a drug screening platform. PDO models also function as valuable models to study tumor evolution. Lee and colleagues have established a PDO bank for bladder cancer and revealed that generally constant truncal mutations with variation in subclonal mutations appear during passaging (19).

In RCC, PDO functions as a model for drug screening and thus guides the selection of effective therapeutic agents. Bolck and colleagues established a biobank of patient-derived 3D ccRCC model, showing a high correspondence to parental tumor and recapitulation of intra- and inter-tumoral heterogeneity, determined by extensive DNA sequencing (20). Furthermore, Fendler et al. characterized and isolated cancer stem cells (CSCs) in ccRCC, which determine the progressiveness of the tumor, and applied CSCs to cultivate ccRCC PDO models. They also validated the significance of the WNT and NOTCH signaling pathways mediating the growth of PDO (21). Na et al. established a concrete protocol of PDO culture directly from surgical resected ccRCC sample. Such PDO preserves the morphology and biomarker expression of parental tumors (22). Grassi et al. also reported an approach to establishing and passaging normal kidney organoids and RCC PDO from surgically resected tissues. They also achieved the transformation between PDO and PDX, which not only guaranteed the long-term establishment of PDO but also paved the way for investigating tumor evolution in RCC (23) (**Table 1**).

**Table 1:** Published articles on establishment of patient-derived organoids of renal cell carcinoma.

Histological type	Tissue collection	Establishment	Success rate	Maximum passage	Drug screening panel	Reference
ccRCC	Surgical specimen	3D patient-derived cells	26/35 (74%)	N/A	N/A	(20)
ccRCC	Surgical specimen	Cancer stem cells	41/55 (74%)	N/A	N/A	(21)
ccRCC	Surgical specimen	Matrigel submerged tumor cells	N/A	N/A	N/A	(22)
Normal tissue						
ccRCC	Surgical specimen	Matrigel submerged tumor cells	10/15 (67%)	15	Sunitinib	(23)
Normal tissue			13/13 (100%)	15	Temsirolimus	
ccRCC	Surgical specimen	Matrigel submerged tumor cells	15/20 (75%)	15	Sunitinib	(24)
					Axitinib	
					Pazopanib	
					Sorafenib	
					Cabozantinib	
ccRCC	Surgical specimen	Minced tissue in type I collagen matrix ALI system	15/26 (57%)	4	Nivolumab	(25)
pRCC			3/3 (100%)			
chRCC			1/1 (100%)			
Wilms’ tumor			1/1 (100%)			
ccRCC	Surgical specimen	Minced tissue in type I collagen matrix ALI system	20/26 (77%)	3	Nivolumab	(26)
pRCC			4/5 (80%)		Cabozantinib	
chRCC			0/1 (0%)			
UC			7/8 (88%)			
Oncocytoma			1/3 (33%)			

ccRCC, clear cell renal cell carcinoma; pRCC, papillary renal cell carcinoma; chRCC, chromophobe renal cell carcinoma; UC, urothelial carcinoma; ALI, air–liquid interface. N/A, Not applicable.

## Tumor Microenvironment in Renal Cell Carcinoma Treatment

In patients with advanced RCC, systemic therapy should be initiated. However, RCC does not show a favorable response to chemotherapies. Recent advances in the molecular mechanism of RCC, especially the inactivation of von Hippel–Lindau (VHL), paved the way for the identification of systemic treatment targeting the tumor microenvironment (TME) (27). Research revealed that the inactivation of VHL contributes to decreased ubiquitin-mediated degradation of a subunit of heterodimeric hypoxia-inducible factor (HIF) transcriptional factor (28). Constitutively accumulated HIF complex enhances the expression of downstream genes, especially vascular endothelial growth factors (VEGF), which leads to the angiogenesis of RCC. Consequently, a specific HIF-2a inhibitor MK-6482 had favorable performance in a recent phase I/II clinical trial (29). Multitargeted, small molecular tyrosine kinase inhibitor (TKI) targeted VEGF, and platelet-derived growth factor (PDGF) such as pazopanib and sunitinib has been proven effective as adjuvant therapy in several clinical trials (30–32). Also, bevacizumab, a monoclonal antibody for VEGF, showed clinical efficacy in metastatic RCC (32). Upon understanding the role of immune escape mechanisms in tumor proliferation and invasion since 2013, several immune checkpoints such as PD-1, PD-L1, and CTLA-4 have emerged as targets to reverse immune exhaustion in TME and counteract negative consequences (34). Such immune checkpoint blockades (ICBs) also benefit patients with advanced RCC. Nivolumab, like a monoclonal antibody for PD-1, has demonstrated a survival benefit in randomized controlled clinical trials (RCTs) (35). Another PD-1 antibody, pembrolizumab, also improves progression-free survival (PFS) of patients with advanced RCC in combination with axitinib compared with sunitinib as a single drug (36).

As illustrated above, unlike a range of solid tumors, systemic therapies of RCC mainly target TME, rather than the malignant epithelium. Consequently, it is necessary to review the mechanisms of TME mediating proliferation and invasion of RCC, which provides precision medicine with targets. Finally, we can work out the importance of integrating TME in PDO as prognostic models and tools for drug screening.

### Hypoxia

HIF is composed of one α subunit with three isoforms (HIF-1α, HIF-2α, and HIF-3α) and one β subunit with two isoforms (HIF-1β and HIF-2β). The β subunit of HIF is constitutively expressed, while the α subunit is induced under hypoxia and dimerizes with the β subunit to form a complex, promoting the transcription of target genes (37). However, the isoforms of the α subunit play distinct but also overlapping roles during hypoxia response. HIF-1α preferentially induces apoptotic and glycolytic pathways, while HIF-2α promotes growth, cell proliferation, and angiogenesis. In many types of solid tumors, both HIF-1α and HIF-2α mediate tumorigenesis and are associated with poor prognosis (38). However, in ccRCC, HIF-2α has tumorigenic activity, whereas HIF-1α functions as a tumor suppressor (39). HIF-2α is also proven as a potential therapeutic target for ccRCC (40).

### Angiogenesis

VHL gene was originally described as the gene responsible for VHL syndrome, a condition associated with an increased risk of retinal angiomas, hemangioblastomas, and ccRCC (41). Based on advanced knowledge of molecular pathways, genetic alterations in VHL were identified as an essential initiator of the tumorigenesis of ccRCC *via* promoting angiogenesis. Actually, it has been reviewed that up to 90% of sporadic ccRCC have the presence of abnormal VHL function (42). VHL proteins complex with Elongin B, Elongin C, and Cul2, which are components of an E3-ubiquitin ligase complex responsible for the proteasome degradation of two subunits of HIF: HIF-1α and HIF-1β. As the result of insufficient degradation, impaired function of VHL will lead to the accumulation of HIF and upregulated transcription of downstream effector genes, such as VEGF, PDGF, erythropoietin, and transforming growth factor (TGF), which play a crucial role in angiogenesis and tumorigenesis (43).

### Immune Cell Infiltration

Historically, the systemic therapy of RCC is initiated by cytokine-based immunotherapy. Interleukin-2 (IL-2) and interferon-α (IFN-α) were considered standard therapy for advanced RCC for a long time (44); even a very small number of patients with advanced RCC have complete responses (CRs) under high-dose IL-2, which is attributed to the mobilization of immune effector cells and the relatively increased number of natural killer cells and CD8+ T cells (45). Among solid tumors, RCC ranks among the highest infiltration of immune cells, with predominantly T cells (50%), followed by tumor-associated macrophages (TAMs, 25%), natural killer (9%), B cells (4%), and other cells (46). However, tumor-infiltrating T cells, different from T cells in normal kidney tissue, are mainly composed of CD8+ T cells with high expression of co-inhibitory receptors such as PD-1 and low levels of proliferation marker Ki-67, which indicate an immune exhaustion state. Moreover, CD8+ T cells in RCC prove to be in a metabolic impaired state with reduced glucose uptake and mitochondrial function, worsening the immune exhaustion state of RCC (47). The immune checkpoint signaling pathway physiologically expressed in normal tissues functions as an inhibitory or stimulatory signaling transducer to protect tissue from autoimmune attack. However, cancer cells evade the immune system and enhance the immune exhaustion state of TME *via* overexpression ligands or receptors of the immune checkpoint. Consequently, ICBs targeting PD-1, PD-L1, and CTLA-4 have shown great effect in reversing immune exhaustion and modulating the metabolism state of immune cells in RCC.

## Patient-Derived Organoids Recapitulate Tumor Microenvironment

RCC is a complex and highly heterogeneous cancer. As a result, treatment responses vary from patient to patient. Nowadays, systemic treatment choices are largely dependent on Memorial Sloan Kettering Cancer Center (MSKCC) scoring (48), suggested by guidelines while lacking biomarkers or prognostic models to tailor treatment plans to individual patients. However, the advances in PDO during the last two decades shed light on the precision medicine of RCC. PDO as a model preserving majority of the characteristic of patients’ tumors is promising in predicting the drug response of individuals. Kazama and colleagues found that RCC PDO models exhibited different responses to TKIs, including sunitinib, pazopanib, cabozantinib, axitinib, and sorafenib. However, responses to PDO require further validation of clinical data (24). A recent prospective clinical study demonstrated the predictive value of PDOs for irinotecan-based chemotherapy in metastatic CRC (49). Wang et al. verified that the drug screening test in PDO models was consistent with clinical efficacy in patients with intrahepatic cholangiocarcinoma (50).

Recent guidelines of RCC stress systemic treatment manipulating TME (51). As illustrated above, multiple targets TKIs inhibit angiogenesis by disturbing the signaling transduction of VEGFR or PDGFR, while ICBs target tumor-infiltrating CD8+ T cells and reverse the immune exhaustion state, both of which modulate TME of RCC instead of tumor epithelium. However, the first-generation PDOs contain exclusively malignant epithelium but impaired TME, thus exhibiting poor performance in predicting clinical outcomes, which hinders the identification of treatment-sensitive patients *via* drug screening based on PDO models and the discovery of predictive biomarkers of drug response (52). Consequently, it is necessary to establish novel PDO models that robustly recapitulate the TME, including immune cell infiltration and interaction with tumor cells, cancer-associated fibroblast (CAF) infiltration, angiogenesis, and extracellular matrix.

### Co-Culture

To overcome the lack of immune cell infiltration in first-generation PDOs, thus establishing a drug screening model of ICB and investigating the interactions between immune cells and tumor epithelium, co-culture with immune cells was developed. A few studies have shown promising results. Dijkstra and colleagues obtained tumor-reactive T cells from the co-culture of PDOs from CRC and non-small cell lung cancer (NSCLC) with peripheral blood lymphocytes (PBLs) (53), indicating that the co-culture system can be used to establish individualized PDO models to study immune therapy and interactions between tumor-infiltrating lymphocytes (TILs) and tumor epithelium. In other cancer types, such as melanoma, breast cancer, pancreatic cancer, and lung cancer, PDO co-culture with PBLs or peripheral blood mononuclear cells (PBMCs) has been a feasible platform to study personalized immune therapy responses (54–57). However, the co-culture system has not been widely applied in the establishment of RCC PDOs. Since RCC presents high immune infiltration, and immune therapy is an essential component of systemic treatment of RCC, it is worthwhile to develop a co-culture system in RCC PDO as a model for drug screening or as a model for investigating the interaction between tumor epithelium and infiltrated immune cells. Recently, Rausch et al. developed a 3D spheroid co-culture system of RCC cell lines and immune cells isolated from PBMCs and recapitulated the responses of drug combinations (58). However, the RCC cell lines are homologous, without the representation of inter-tumor heterogeneity. Grassi et al. successfully established PDO in patients with RCC, which preserved the expression of PD-L1 and PD-L2, suggesting a promising application in a co-culture system (23). Consequently, generating PDO models of RCC co-culture with immune cells is substantial and possible in the near future.

### Air–Liquid Interface

As illustrated above, RCCs have high immune infiltration and present substantial heterogeneity, which contributes to the difficulty in the prediction of drug responses and creates exigency for establishing PDO models recapitulating the patients’ situation as closely as possible for studying personalized medicine. However, co-culture with PBMCs or TILs, albeit preserving immune cells in TME, fails to preserve the diversity of immune cell types in TME, which proves essential in drug responses. Moreover, the physical architecture of TME is also disturbed in the process of co-culture. Consequently, a novel generation of organoids based on the air–liquid interface (ALI), which closely resembles the *in vivo* situation, has been developed and applied as a preclinical tool for the investigation of several diseases (25, 59, 60). ALI-PDO method successfully preserves the complex histological TME architectures *via* mechanically mincing, rather than dissociating tissue with collagenase, which is commonly applied in the first generation. The addition of IL-2 in the medium also plays a central role in preserving the viability of CD3+ TILs (25). The ALI methodology was initially introduced into the culture of murine intestinal organoids to maintain mesenchymal cells and supply paracrine signaling (61–63). Until 2018, Neal and colleagues optimized protocols for establishing the ALI-PDO model in a series of surgically resected tumors, including colon adenocarcinoma, bile duct ampulla adenocarcinoma, lung adenoma, and renal clear cell carcinoma. At the histological level, ALI-PDO accurately presents the heterogeneity and architecture of primary tumor with retention of stromal, CAFs, and diversity of immune cell population. At the gene level, scRNA-seq shows a high concordance in TCR between ALI-PDO and RCC tumors. Moreover, ALI-PDO has been proved to confidently recapitulate the effect of PD-1/PD-L1-dependent immune checkpoint (25). The team of Neal also developed a method for determining the responsiveness of ALI-PDO to immunotherapeutic agents by measuring mRNA or protein markers associated with immune activation, which paved the way for utilizing ALI-PDO as an immunotherapeutic drug screening model (25). Two years later, Esser and colleagues applied the protocols of Neal et al. to cultivate ALI-PDOs from renal tumors and test drug efficacy. ALI-PDOs from RCC showed heterogenic responses to target therapy (TKI) and ICB, which is in line with the clinical situation. By applying this model, researchers also recapitulated that responses of nivolumab are dependent on CD8+ T-cell infiltration, rather than the expression level of PD-L1 in tissue (26, 64). This study provides the perspective of ALI-PDO in functioning as a preclinical model for tailoring RCC treatment plans. Moreover, Vilgelm and colleagues reported a protocol of PDO cultivation based on fine-needle aspiration (FNA), which is adapted to drug screening. They applied Wnt3A and IL-2-containing medium to effectively preserve the viability of immune cells in RCC PDO (65). Vilgelm et al. demonstrated the ability of predicting clinical outcomes before initiating systemic treatment of RCC patients by cultivating PDOs from diagnostic FNA (65). All of these studies show that ALI-PDO is a promising preclinical model, reliably recapitulating both heterogeneity and architecture of TME, which has the ability to predict responses of first-line treatment of RCC, including TKIs and ICBs, and in turn guide personalized medicine.

### Tissue Slice Culture

To retain the heterogeneity and architecture of TME in RCC individualized models, patient-derived tissue slice culture (PDTSC) has been promoted. The first PDTSC for RCC was prompted by Weissinger and colleagues in 2013, functioning as a model to study the oncogenic signaling pathway of RCC (66). Martin and colleagues refined *ex vivo* cultivation procedures of PDTSC for hepatic metastatic CRC (67), which was utilized by Stenzel et al. to examine the effect of nivolumab in RCC by monitoring TILs. Investigators revealed that nivolumab-mediated reduction in PD-1 expression and altered activation status of TILs, especially CD8+ T cells, are indicators for responses to ICBs (68). Roelants et al. also developed a PDTSC model for RCC to evaluate treatment responses (69). Slice culture from RCC showed perfect consistency with parental tumor both histologically and genetically, which had the ability to evaluate the cytotoxic effect of targeted therapies. By applying PDTSC models, CD8+ T cells were predicted as markers indicating immunotherapy responses, which is in line with contemporary research (70).

Here, we concluded the characteristics of PDO models and differences between novel generation PDO models in **Table 2**.

**Table 2:** Comparisons between conventional and next-generation patient-derived models of renal cell carcinoma.

		First-generation PDO	PDO plus		
			Co-culture	ALI	TSC
Histological characteristics		Preserved	Preserved	Preserved	Preserved
Genetic alteration		Preserved	Preserved	Preserved	Preserved
Component of TME	ECM	−	−	+	+
	Immune cells	−	+	+	+
	CAFs	−	−	+	+
Architecture of TME		−	−	+	−
Availability of live cell analysis		+	+	+	−
Testable drug classes		TKIs	TKIs; immunotherapy	TKIs; immunotherapy	TKIs; immunotherapy
Reliability as preclinical model		+	++	+++	++

PDO, patient-derived organoid; ALI, air–liquid interface; TSC, tissue slice culture; TME, tumor microenvironment; ECM, extracellular matrix; CAFs, cancer-associated fibroblasts; TKI, tyrosine kinase inhibitors. #-, does not have or not available; +, perform fine; ++: perform very well; +++: perform excellent.

## Discussion

PDO is a reliable and economical model for drug screening for various cancer types. The results of drug screening not only indicate clinical treatment choice but also can be used to explore predictive biomarkers of drug responses. In this review, we summarized recent advances in the establishment of PDO models in patients with RCC. Unfortunately, the majority of reported culture methods remain the first generation PDO, which lacks the infiltration of the TME. However, the main targets of systemic treatment in RCC are neo-vasculature and infiltrating immune cells. Consequently, it is urgent to develop PDO models with preserved TME in patients with RCC. In other cancer types, co-culture, ALI, and TSC have been extensively applied to recapitulate the microenvironment. In the future, more effort should be put into the integration of such methods in RCC PDO. Moreover, the peripheral immune system, such as circulating immune cells and peripheral lymph nodes, contributes to the responses of immune therapy (71). Chimeric antigen receptor redirected T (CAR-T) cells also show effects on solid tumors (72). Consequently, in the near future, the interaction between cancer epithelium and peripheral immune system cannot be ignored, especially in RCC, a tumor with highly infiltrated immune cells, which means more holistic models, and integrating systemic conditions in PDO will attract more interest. For example, organ-on-a-chip models highly mimic the physical condition by seeding multiple cell types of the human organ into engineered chambers with perfusion, which provides new perspectives for the investigation of a holistic response to the drug (73). Recently, organ-on-a-chip models are mainly based on microfluidic devices (74). With the development of organoids, “organoids-on-chip” will also appear to recapitulate *in vivo* environment more exactly.

In conclusion, PDO as an essential tool for personalized medicine goes through an evolution during the past few years, with a more accurate recapitulation of *in vivo* conditions. As a result of highly infiltrated immune cells in RCC, progress in mimicking the RCC TME is still needed in the development of PDO.

## Author Contributions

BW and YX drafted the article, and WZ revised it critically for intellectual content. All authors contributed to the article and approved the submitted version.

## Funding

This study was supported by the National Natural Science Foundation of China (Nos. 82173214 and 81972369).

## Conflict of Interest

The authors declare that the research was conducted in the absence of any commercial or financial relationships that could be construed as a potential conflict of interest.

## Publisher’s Note

All claims expressed in this article are solely those of the authors and do not necessarily represent those of their affiliated organizations, or those of the publisher, the editors and the reviewers. Any product that may be evaluated in this article, or claim that may be made by its manufacturer, is not guaranteed or endorsed by the publisher.
